# Melanin, the What, the Why and the How: An Introductory Review for Materials Scientists Interested in Flexible and Versatile Polymers

**DOI:** 10.3390/polym13101670

**Published:** 2021-05-20

**Authors:** A. Bernardus Mostert

**Affiliations:** Department of Chemistry, Swansea University, Singleton Park, Wales SA2 8PP, UK; a.b.mostert@swansea.ac.uk

**Keywords:** eumelanin, bio-macromolecule, polymer, poly-indolequinone, biomimetic

## Abstract

Today, western society is facing challenges to create new medical technologies to service an aging population as well as the ever-increasing e-waste of electronic devices and sensors. A key solution to these challenges will be the use of biomaterials and biomimetic systems. One material that has been receiving serious attention for its biomedical and device applications is eumelanin. Eumelanin, or commonly known as melanin, is nature’s brown-black pigment and is a poly-indolequinone biopolymer, which possess unique physical and chemical properties for material applications. Presented here is a review, aimed at polymer and other materials scientists, to introduce eumelanin as a potential material for research. Covered here are the chemical and physical structures of melanin, an overview of its unique physical and chemical properties, as well as a wide array of applications, but with an emphasis on device and sensing applications. The review is then finished by introducing interested readers to novel synthetic protocols and post synthesis fabrication techniques to enable a starting point for polymer research in this intriguing and complex material.

## 1. Introduction

Advanced health informatics is one of the 14 grand challenges set out by the United States National Academy of Engineering for the 21st century [1]. It recognizes the need for medical devices for servicing an aging population. Major pharmaceutical companies such as GlaxoSmithKline are also researching new ways of doing electrical based medicine termed “electroceuticals” [2]. A parallel development for modern society is the increased use of devices for sensing, but with this need has come the concomitant increase in electrical and electronic waste [3]. This has become a significant problem not only due to the sheer volume of waste (45 megatons per year) but also the fact that the waste can be hazardous to the environment [3]. Indeed, there is a growing need to develop “green electronics”, i.e., to make device materials more eco-friendly and to make devices with a lower energy input [4].

A key solution to both the above societal demands will be the development of bio-inspired materials. Nature is an excellent teacher, making available a wide variety of materials and methods to take inspiration from and to apply for use [5]. One class of compounds that are of interest for application are the poly-indolequinones, of which the two main species are the polydopamines and the melanins, not counting their derivatives. Polydopamine is a functional coating that has received much attention [6] and melanin is better known as the human skin pigment [7].

These materials are polymeric and with recent advances have opened up potential avenues for using them in flexible and versatile setups including situations that utilize their conductive properties.

This review focuses on melanin, or more specifically, eumelanin. There are currently a couple of reviews available in the literature on melanin, which focus on various topics and will be referenced later on. However, this current work’s aim is to introduce interested polymer and other materials scientists to the basics of the material, why it is interesting, and if sufficiently induced, to give information on how to practically start working on the material. All of the above will be within a theme of utilizing melanin as a flexible, versatile and conductive material that can be applied in a wide variety of settings, but with some emphasis on device or sensing applications.

As such, the review is structured as follows. The first section essentially deals with the “what” of melanin. What it is made of and what the current understanding is about its structure. This section is followed by the “why” of melanin. Why is melanin an interesting material to study? This will cover basic physical and chemical properties of the material, which in this author’s opinion is inherently interesting, and also how melanin is being applied as an advanced functional polymeric material. Finally, the article will discuss the “how”. The hope is that any readers new to melanin would find the material interesting to study, but may not know exactly where to start. This “how” section aims to provide a starting point. As such, this review is not envisaged to be exhaustive, but to act as the metaphorical kindling to research interest, since there is much opportunity in this complex polymer [8], and to highlight some of the outstanding issues regarding nature’s most ubiquitous pigment [9].

## 2. What Is Melanin?

Mention of melanin was first made by Aristotle, where he discusses the mollusk species sepia, which is known to discharge a black pigment (melanin) to ward of predators [10]. This source of melanin is considered the standard form of natural eumelanin [11,12]. Indeed, the name “melanin” is derived from the Greek word for black, μελαζ.

The melanins are a class of compounds, defined as pigments, of diverse structure and origin derived by the oxidation and polymerization of tyrosine in animals or phenolic compounds in lower organisms [11]. Generally, within this class there are three main compounds, though this could be extended to five compounds if considering non-nitrogen containing systems [13]. Keeping with the more traditional, nitrogen containing materials, the first compound is called eumelanin and is what is usually associated with the term melanin. It is an insoluble black-brown pigment, derived at least in part from the oxidative polymerization of L-dopa via 5,6-dihydroxyindole intermediates [11]. There is pheomelanin, a yellow-to-reddish brown sulfur containing pigment derived from the oxidation of cysteinyldopa precursors via benzothiazine and benzothiazole intermediates [11], which is recognizable as the pigment present in red-haired individuals. Finally, neuromelanin is a dark pigment produced within neurons by the oxidation of dopamine and other catecholamine precursors [11]. This latter compound is found in the *substantia nigra* of the brain stem [14] and is a material that has been shown to contain a pheomelanin core and a eumelanin outer shell [15].

In this review the focus will be on eumelanin, the brown black pigment. It is the most common form, and can be considered the archetypal melanin that has the most materials research associated with it. As is standard in the rest of the literature, the terms melanin and eumelanin will be used interchangeably for the rest of the manuscript unless specified otherwise.

### 2.1. Melanin Monomers

The ability of melanin to reduce and oxidize other molecules is, to a large extent, determined by the redox properties of its monomer units [7,16]. As such, it is useful to have a brief overview of these monomers/moieties that are involved in the synthesis and structure of melanin.

**Figure 1:**
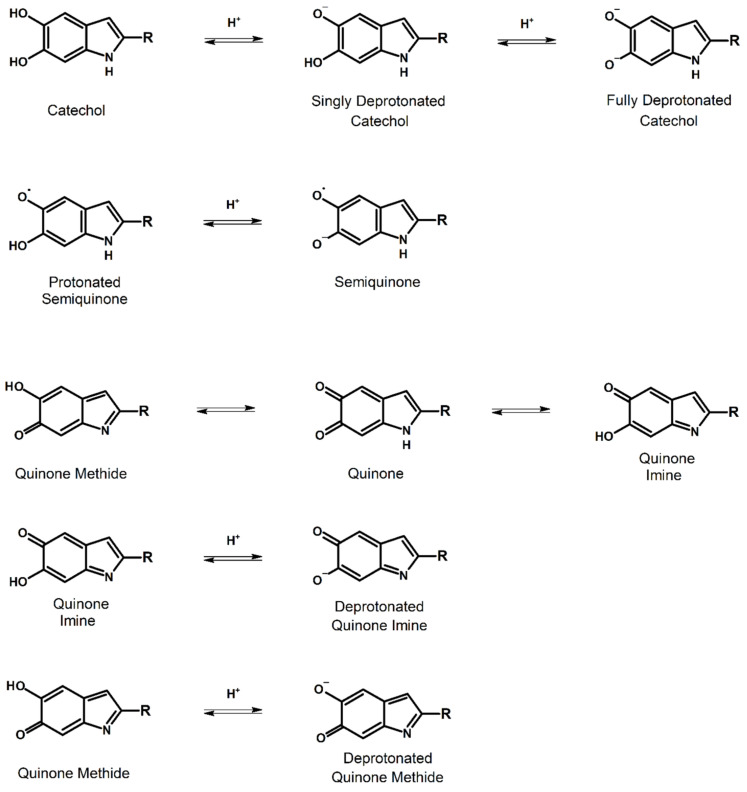
The starting monomers of melanin and their various redox states and tautomers including their deprotonated states. (i) For the catechol where R = H, one has a 5,6-dihydroxyindole (DHI). For R = COOH one obtains a 5,6-dihydroxyindole-2-carboxylic acid (DHICA). These two molecules are the primary starting monomers for melanin synthesis. This figure was adapted from Chemical Physics, 546, A.B. Mostert, On the free radical redox chemistry of 5,6-dihydroxyindole, 111158, Copyright Elsevier (2021) [17].

Melanin is produced primarily from two key monomers, 5,6-dihydroxyindole (DHI) and 5,6-dihydroxyindole-2-carboxylic acid (DHICA) and their various redox and tautomeric states, which are depicted in Figure 1.

The first thing to note is that the starting monomers are *ortho*-hydroquinones or catechols, and they behave as such [17,18,19,20]. Furthermore, these monomers are highly reactive [18,19,20,21]. The most reactive sites appear to be the 2, 4 and 7 positions, at least for DHI [22]. Furthermore, the catechols are also sensitive to oxygen, and can undergo auto-oxidation under mild conditions [19].

The presence of a free radical within melanin is not surprising [7] given that it is based upon an *ortho*-quinone system and that much of the original observed behavior is in line with that of semiquinone moieties [23,24,25,26]. However, an important observation is noted here, standard *ortho*-quinone semiquinones are very unstable, however, for the case of melanin the related melanin subunits are quite stable and exhibit substantially lower reactivity. This is well demonstrated by the direct detection of melanin in dinosaur fossils, including an electron paramagnetic resonance experiment [27]. The lower reactivity is probably due to their restricted accessibility as a result of intramolecular interactions and steric hindrance [7]. However, as a monomer entity, the semiquinone can be formed by utilizing pulsed radiolysis [28,29], with a pK_a_ of 6.8, which is higher than for corresponding *ortho*-benzoquinone radicals [28] and has a fairly long lifetime extending into the hundreds of microseconds [29].

**Figure 2:**
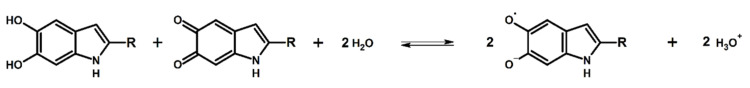
The comproportionation reaction. Two monomers/moieties of different oxidative states react to yield an intermediate oxidative state (the semiquinone). This figure was adapted from Chemical Physics, 546, A.B. Mostert, On the free radical redox chemistry of 5,6-dihydroxyindole, 111158, Copyright Elsevier (2021) [17].

The importance of the semiquinone must not be discounted, since its formation via a comproportionation reaction (Figure 2) is key to the electrical properties and hence device applications of melanin.

The quinone monomer, is a most elusive monomer, which is believed to exist as a moiety within melanin. In the monomer state, it is highly reactive and very difficult to detect, with many results based upon inference [21]. For example, work by Edge et al. was able to form an oxidized product from the semiquinone where an isosbestic point was observed with pulsed radiolysis experimentation [29], which is consistent with quinone formation. However, this is not a direct observation since the results could also indicate a tautomeric form, and as such, there has been much speculation about its existence [21,30]. However, a closely related analogue has been observed alongside its corresponding semiquinone [30], indicating that the quinone should certainly exist as a system within the polymer as connected moieties.

Coming to the tautomers of the quinone, the existence of tautomers within melanin was first proposed in 2002 based upon DHI/melanin materials [31]. In this article, the authors suggested that the quinone imine is a major component of the melanin. However, subsequent computational studies have all indicated that the quinone methide is more stable than the corresponding imine [30,32], though less stable than the quinone. It appears that it is now more generally accepted that the quinone methide is the preferred tautomer, in line with other *ortho*-quinone systems [18,19,20]. What is interesting to note is that the methide appears to be a key intermediate in DHI and DHICA formation in the early stages of melanogenesis [19,20].

### 2.2. Melanogenesis

**Figure 3:**
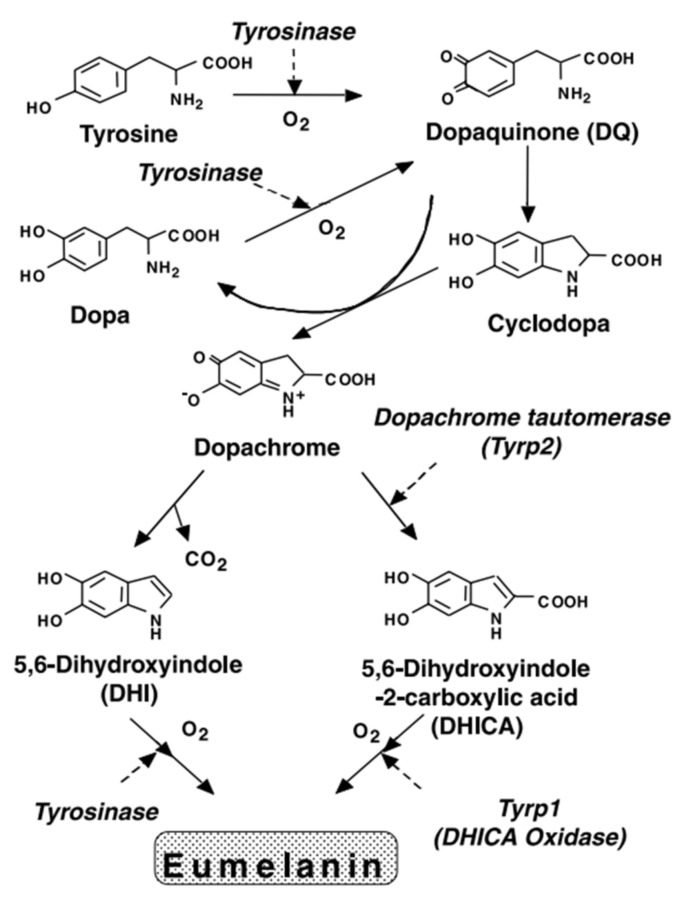
The Rapor–Mason scheme for melanogenesis. Adapted with permission from [35]; © 2003 John Wiley & Sons, Inc. (Hoboken, NJ, USA).

Melanogenesis does not have direct application to the material scientist, but it may prove instructive as to the kind of chemistry that is involved in synthesizing melanin. As such, a short overview is given here. Melanin has its origins, at least within humans, within the melanocytes. It follows the famous Rapor–Mason scheme (Figure 3) [33,34,35], which has captured most of the early stages of melanogenesis.

First, tyrosine is oxidized by tyrosinase to form dopaquinone as an intermediate product. Dopaquinone then undergoes intramolecular addition of the amino group yielding cyclodopa (also known as leucodopachrome). Redox exchange between cyclodopa and the dopaquinone can then yield two products: dopa and dopachrome (an orange red pigment readily observed by eye at the early stages of synthesis if done synthetically). Dopa can be oxidized (with the help of tyrosinase) to reform dopaquinone whereas the dopachrome decomposes to yield mostly DHI, and to a lesser extent, DHICA [35].

**Figure 4:**
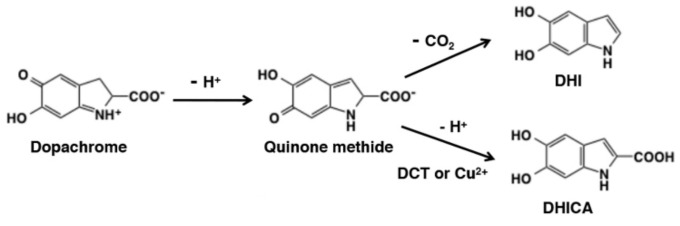
The proposed mechanism of DHI and DHICA from dopachrome via the quinone methide intermediate. In the formation of DHI, the acid group is spontaneously lost as carbon dioxide whereas with the help of either Cu^2+^ or dopachrome tautomerase (DCT), the acid group can be kept intact to form DHICA. Figure adapted with permission from Ito et al. under Creative Commons Attribution—Published by MDPI AG [18].

The formation of DHI from dopachrome has been demonstrated to occur via the quinone methide discussed above, the latter being reduced (see Figure 4) [18]. Presumably something similar occurs for DHICA, but has not been formally demonstrated [18].

When it comes to the formation of melanin proper from DHI and DHICA, current knowledge is much less clear. However, some progress has been made, famously by Italian chemists. For example, Edge et al. demonstrated that dopaquinone can react with DHI to form the DHI quinone, a highly reactive system that goes on to form the black insoluble polymer [29]. This may not hold for DHICA per se, which may need enzyme intermediates [18]. DHI quinone and DHICA quinone can readily dimerize with either DHI, DHICA, or both, to yield homodimers and heterodimers. Furthermore, various oligomers up to the stage of tetramers have been isolated and characterized in the form of acetylated derivatives for DHI or non-derivative forms of DHICA [36]. These oligomers form supposedly through free radical polymerization, but the exact mechanism to date is still up for debate [22].

The existence of these oligomers is of great importance, since they lead to melanin’s supramolecular structure, which will be covered in the next section. However, before doing so, a note needs to be made about melanogenesis. The basic mechanisms discussed above for the in vivo case only considers the formation of eumelanin and as such is really an idealization, since the formation of eumelanin in an organism is invariably accompanied with the formation of pheomelanin in something termed mixed melanogenesis [37]. For real world synthesis in vivo, both the formation of eu- and pheomelanin need to be considered together.

When it comes to synthetic methods, the usual aim is to mimic the above conditions to form a biomimetic material, with attention being paid to the type of precursor, reaction conditions (such as oxidants, pH, medium and reaction time) and post synthetic processing [11]. An excellent article to consult is d’Ischia et al., listing the type of precursors and conditions to use [11].

More novel techniques will be covered below in the “How” section, more suited to making melanin analogues for use in materials applications. These methods may or may not create true biomimetics, but will yield materials with similar properties.

### 2.3. Melanin’s Structure

Melanin’s supramolecular structure, has had some controversy surrounding it [7]. The main reason for this is that melanin is not a traditional polymeric compound. Rather, it is a disordered and amorphous material [7,11,38,39] that is insoluble in most common solvents, making standard characterization techniques untenable [7,11].

However, multiple works across the years, utilizing a wide array of differing techniques, have made much progress towards understanding melanin’s structure, even though the underlying explanation for their exact origins are still up for debate [22]. Multiple techniques, including X-ray diffraction studies [40,41], transmission electron microscopy [42,43], scanning tunneling microscopy [44,45] and atomic force microscopy [46], have established that melanins tend to form oligomeric structures of 3–5 monomer units [7] that then stack on one another via the π–π interaction, à la Hunter and Sanders [47].

These stacked oligomeric structures, which have a density of 1.37 g cm^−3^, are of the order 1–5 nm large, are stable and difficult to break apart [40,41]. Indeed, only partial solubilization can be achieved at alkaline pH [48].

The formation of oligomers has been mentioned above, but the modelling and understanding of these molecules have had a long history, dating back to at least the early 2000s [46,49,50], with ideas of porphyrin like structures also being suggested [51,52,53].

**Figure 5:**
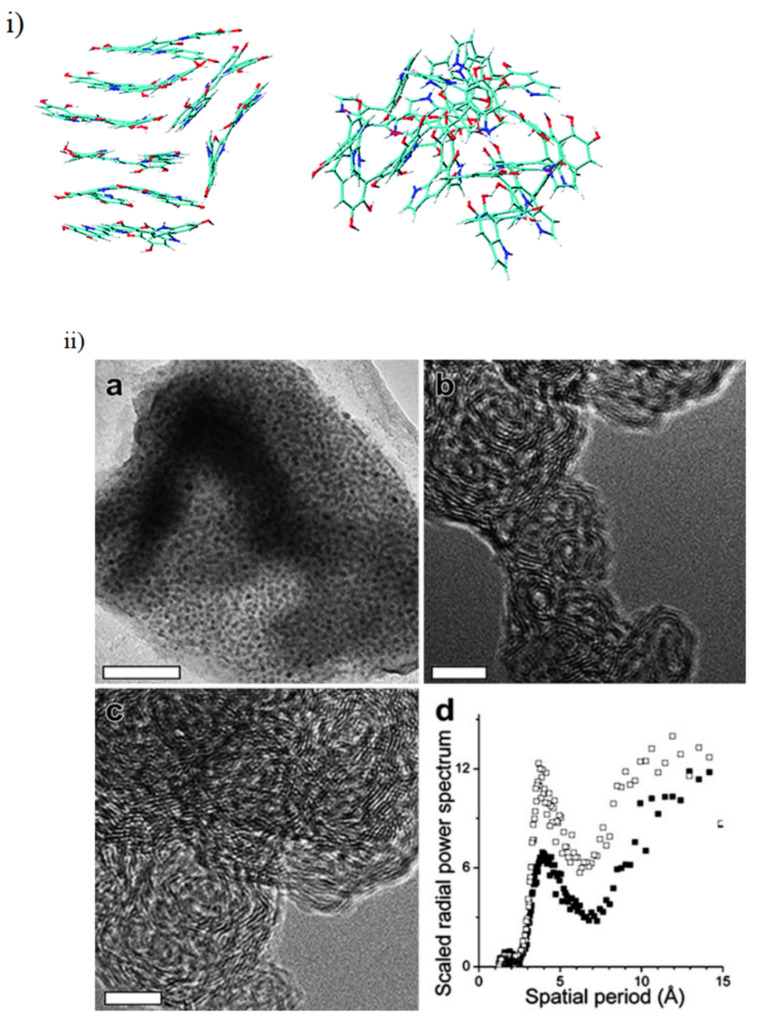
(**i**) A model stacked oligomeric structure proposed for melanin. Figure adapted with permission from C. Chen, F. J. Martin-Martinez, G. S. Jung and M. J. Buehler, *Chem. Sci.*, 2017, 8, 1631 under Creative Commons Attribution—Published by The Royal Society of Chemistry [22]. (**ii**) Real space images from TEM showing the stacking of the oligomers in both synthetic biomimetic versions and natural sources of melanin are also depicted. (**a**) Depicted is a low-resolution bright field transmission electron micrograph of synthetic melanin (scale bar: 50 nm). (**b**) A high-resolution bright field transmission electron micrograph of synthetic melanin (scale bar: 5 nm). (**c**) A high-resolution bright field transmission electron micrograph of synthetic melanin (scale bar: 5 nm). (**d**) A scaled radial power spectrum of (**b**) shown as solid black squares and (**c**) shown as open black squares. The narrow peaks in the spectra are centered at 3.9 A˚ and 3.7 A˚, respectively, for the images (**b**,**c**). Adapted from [42] with permission from The Royal Society of Chemistry.

Recently, a large study by Chen et al. has modelled over 3000 different configurations of DHI oligomers from monomer up to tetramers (not including porphyrin structures), which demonstrates the extreme heterogeneity the monomer building blocks of melanin can have in forming the final polymer [22]. These authors also modelled the potential stacking of the most stable tetramers and found that the molecules do have a tendency to stack as the aforementioned morphological studies suggested (see Figure 5). However, it should be noted that Chen et al.’s main focus was on the reduced monomer of DHI, leaving plenty of scope to envisage the effect of DHICA and the oxidized states of potential moieties.

**Figure 6:**
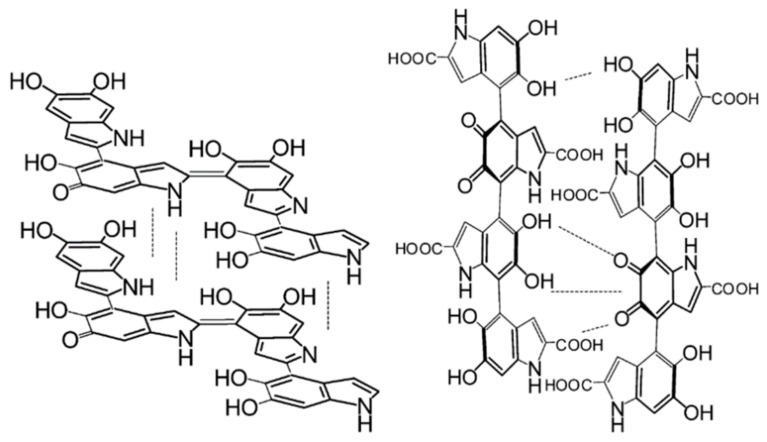
Representative structures of melanins that are DHI (**left**) or DHICA (**right**) based. Reprinted with permission from M. d’Ischia, A. Napolitano, V. Ball et al., *Acc. Chem. Res.*, 2014, 47, 3541–3550. Copyright 2014 American Chemical Society [55].

Given the above, it should be noted that it is known that differing starting ratios of DHI/DHICA can profoundly change the packing of the oligomeric structure within melanin, with the different monomers having distinct affinities for stacking (see Figure 6) [54,55].

This is important since it leads to differences in the structures of synthetic melanins and naturally sourced melanins: the former tend to have a DHICA content of around 10%, whereas naturally occurring materials can have up to 50% DHICA content [55,56,57]. This has implications for the differing redox activities for the differently sourced materials [55], and potentially explains the differences observed in the packing structures (see for example Watt et al. [42]). The intricate details relating to the difference between synthetic and natural melanins is beyond the scope of this review, however, it is sufficient to say that the above information should convince the reader that, at basis, melanin should be considered a stacked oligomeric system, as opposed to a standard long chain polymer. This has profound consequences for the basic properties of melanin and its processing in technological applications.

## 3. Why Research in Melanin?

Having established the broad outlines of melanin as a material, a pertinent question to ask is why does it have such an enduring fascination for scientists? Furthermore, how has the material been used as a flexible polymeric material? The purpose of this section is to lay out to the reader a taste as to why.

### 3.1. Medical Disease Implications and Biological Function

Melanin is a natural material that is synthesized in vivo by various organisms, including human beings. Within humans, it is found throughout the human body including the eyes [58], the inner ear [59], the brain stem [14], hair [60] and of course most famously, the skin [61]. Due to its prominence in the skin and its optical behavior (which we will cover below), melanin is believed to exist predominantly as a protective pigment against UV-radiation [7]. However, due to its location in parts of the body that have nothing to do with light, many have suggested that melanin’s primary function is something else, as humorously captured by Hill [62]. Example ideas counter to the photo protection role are; that melanin plays a stronger role in the immune response [63]; that it is an antioxidant by being a free radical scavenger [64]; that melanin protects against transition metal ion poisoning [14]; that melanin potentially protects against particle radiation [65]; that it is there for pigmentation such as camouflage [66]; that melanin is an energy transducer [39]; and that it acts as a metal ion reservoir and regulator [67]. The aforementioned potential functions cover just those that are relevant to humans. However, given that melanin is found in many other organisms, there is speculation on a plethora of additional potential functions [39].

Indeed, melanin has been associated with disease states that do not seem to be related to one another at all, such as (as could be expected) melanoma skin cancer [61,68], but, interestingly, also Parkinson’s [14]. Clearly, its function cannot be purely photo protection, and indeed, that may not even be its primary purpose.

What makes the above especially vexing, is that melanin breaks the standard rules of structural biology, where a well-defined structure, leads to a well-defined property and hence a well-defined function. As was noted above in discussing the basic material structure of melanin, it has no well-defined structure. This naturally has made assigning a primary function to melanin difficult, which is why melanin as a material with medical implications continues to draw attention. The aim of many a scientist is to understand the structure-property-function relationship and from there to investigate how melanin is involved in disease states and how to potentially treat patients.

### 3.2. Unique Physico-Chemical Properties

While, the above may be interesting, for a materials scientist interested in creating a flexible material for device and sensing applications, the physical and chemical properties of melanin are far more important. These properties are rather unique to the material. Below a short overview if given of the key properties of melanin and their potential origin.

**Figure 7:**
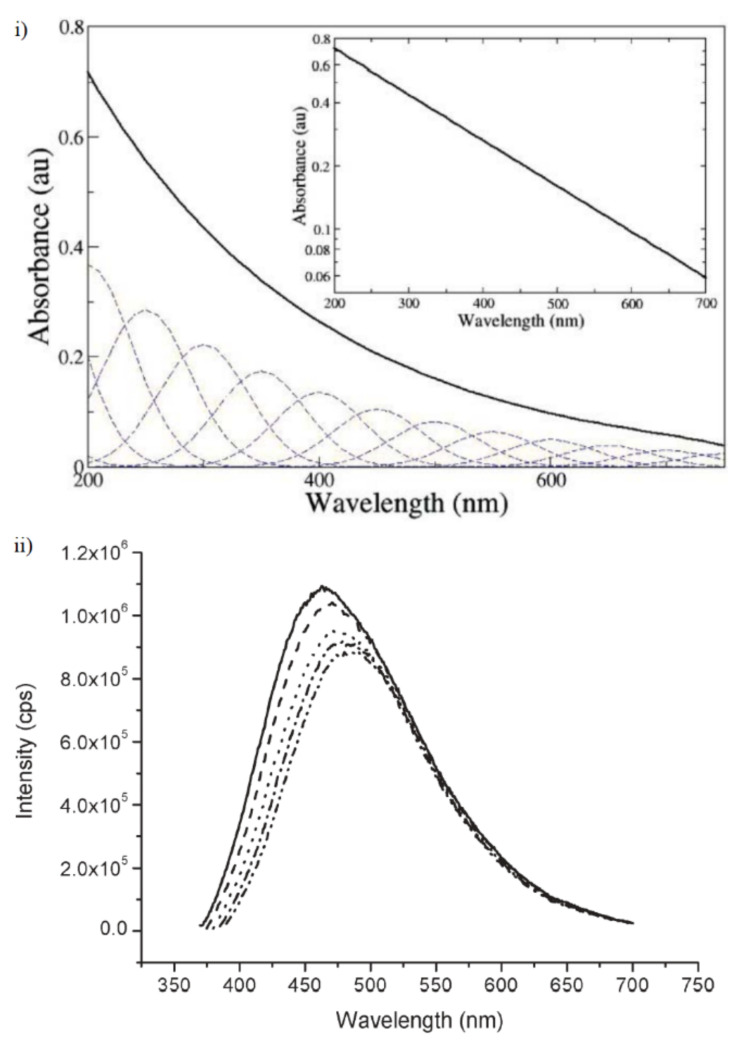
(**i**) An example of melanin’s UV-Vis absorbance spectrum. A log-linear plot of the data is depicted in the inset, demonstrating its exponential behavior. (**ii**) An example of the fluorescence emission spectra of melanin. The various dotted lines indicate the spectra obtained with variation of the laser pump energy (360 nm—solid line, to 380 nm—inner dot–dash line, in 5 nm intervals). Figures adapted from Ref. [69] with permission from The Royal Society of Chemistry.

We first start with melanin’s UV-visible absorbance spectrum. Rather famously, it has a featureless broad band exponential decay profile (Figure 7i), with no peak, which is unusual for an organic chromophore. The dotted overlapping Gaussian lines depicted in Figure 7i are an idealized set of spectra of potential transitions for individual chromophore components within melanin, which should illustrate the fact that the absorbance profile can be explained as summation of multiple peaks [69]. This explanation is based upon the chemical disorder and heterogeneity of melanin [70], a consequence of the reactive nature of the monomer building blocks and stacked oligomeric structure as discussed above.

In contrast to the absorbance spectra of melanin, the fluorescence emission spectrum of melanin (Figure 7ii) clearly shows peaks, but with a change depending on the laser pumping energy used. This contrast between the absorbance and emission is a clear violation of the mirror image rule in organic spectroscopy. However, the behavior of the emission spectra suggests that different individual chromophores within melanin are emitting, which lends support to the idea of the overlapping chromophore absorbance spectra model depicted in Figure 7i. Finally, it should be noted that the emission spectra obtained for melanin has a smaller than 0.1% quantum yield, indicating that melanin dissipates >99.9% of the UV-Vis energy as heat [71], which naturally leads to the conclusion that melanin is an excellent photo protectant against optical and UV radiation and as such explains why it is found in the skin. To bolster this conclusion, Li et al. recently found that melanin’s ability to absorb UV radiation increased after an initial exposure to said radiation [72]. However, it is still not well understood what the coupling, internal conversion mechanism is in how melanin transforms optical energy into heat to such a great extent.

A natural question to ask is: how photostable is melanin? Melanin exhibits variable degrees of photostability [73,74], but the actual photostability of melanin depends on the exact experimental conditions, such as the source of melanin (natural vs. synthetic), hydration state, pH, presence of redox-active metal ions, oxygen concentration, the supermolecular structure of the pigment granules (e.g., see [75]) and how much light intensity is being applied over what time period (see the high intensities and long time periods applied to induce degradation [73,74]). However, in principle, melanins could be suited as an absorbing component for device applications in bioelectronics [39]. A good reference for a discussion on photodegradation of melanin is the review by Xiao et al. [76]. Closely related to the question of photostability is that of solvent stability. As such, the material appears to be stable in most solvents, as will be seen below when it comes to melanin processing techniques. However, as with the photo bleaching, the oxygen concentration, the pH (less stable at high pH) and the presence of redox active metal ions are important factors determining the long-term stability of melanin [7].

What may not be immediately obvious, given melanin’s insoluble nature, is that the material itself is quite hygroscopic. Water adsorption isotherms of melanin have indicated that it can absorb up to 20% of its own weight in water [77,78,79], which corresponds roughly to ~2 water molecules to a monomer moiety. To dry the melanin takes considerable effort, requiring hours of active pumping under vacuum or heat to drive the water off [80]. It has also been recognized that melanin has incorporated within its structure a “hard water” [81], which cannot be removed except via the decomposition of the polymer itself [80]. This basic observation that melanin is hygroscopic and that it holds onto its water strongly has implications for some of its other properties as will be shown below.

**Figure 8:**
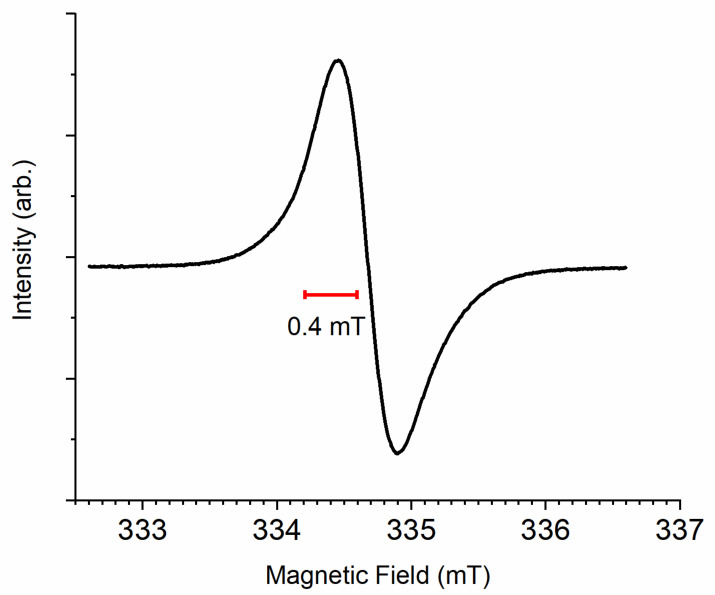
An example electron paramagnetic resonance (EPR) spectrum of melanin. The sample was a solid state, neutral synthetic sample under vacuum. Data used is from Reinecker et al. [89]. Indicated is the width of the spectrum at full width half maximum.

Melanin has a well-known, stable free radical that can be detected by electron paramagnetic resonance, which is a single, slightly asymmetric line [7] (see for example Figure 8). Indeed, melanin was one of the first biomaterials that had a reported free radical signal [82]. Naturally, the presence of a long-lived organic radical, especially within a material that supposedly has a protective function, is naturally puzzling. The behavior of melanin’s radical signal can, at least within solution, be well described by basic *ortho*-quinone chemistry. Essentially, the free radical signal has as its origin a semiquinone radical with the associated comproportionation reaction, demonstrated via the chelation of transition metal ions [23], pH dependence [17,25], Arrhenius temperature dependence [24], photo generation [83] and numerous other sets of reactive behaviors [7,84], with the exception that the radical is stable and long lived. However, there are some key differences and puzzles remaining when it comes to melanin’s radical behavior. With a full width half maximum of around 0.4–0.6 mT at X-band [7], the electron paramagnetic resonance (EPR) signal for melanin is quite broad for an organic radical, but can be explained by the numerous hyperfine couplings to protons on an indole ring [85]. Furthermore, there is a change in the isotropic *g*-value of the signal with pH (2.0034–2.0045) [25], which may be indicative of a change from the phenoxyl form of the semiquinone (i.e., protonated semiquinone) to the anionic form, or may be indicative of other radical species within melanin (see below). In addition, there are questions surrounding the behavior of melanin’s radical signal in the presence of specifically copper ions. Initial work suggested a standard redox reaction between Cu^2+^ ions [86] and the radical that modulates the signal of both, but later it was suggested that no chemical reaction occurs but is instead due to a physical, magnetic interaction [87], which is unusual. However, as with melanin research, this has been brought into question recently [88], leaving open questions for researchers to answer, not just philosophically, but with real material implications. Finally, as pertaining to melanin solutions, the extent of free radical production at high pH is much less than what would be expected for a standard *ortho*-quinone system. This latter observation may be due to tautomer formation in melanin and its moderate pK_a_, leading to a depletion of quinones for semiquinone production [17]. This suggestion has only been inferred from computational modelling but has not been confirmed experimentally.

In the condensed matter phase, melanin’s radical signal brings up more questions than answers to date. The temperature dependence follows more closely a Curie–Weiss dependence [80] and it has a hydration dependence where the signal decreases with intensity [26]. This signal decrease, though, can be further enhanced or moderated depending on pH [26]. Furthermore, the signal can be decomposed into several smaller signals [26,85,90,91], with support experimentally for qualitatively two different radicals when using a combination of pH, microwave power, hydration, and photo excitation with one signal being identified as the anionic semiquinone radical and another whose *g*-value is consistent with a carbon centered radical [92]. The two main parts of the signal may be a superposition of the phenoxyl form and the anionic form, as possibly indicated by the solution work mentioned above [17], but the complexity of the signal and computational work indicates potentially even more radicals [85,90]. There are also differences between synthetic and natural samples and the proportion of DHI to DHICA also affects the signal [54]. As such, the free radical signal in melanin continues to hold a fascination. However, with all that said above, with the appropriate set of experimental parameters, the radical signal in melanin is more or less due to its semiquinone nature, in which the comproportionation reaction (Figure 2) plays a key role. This latter reaction is important in understanding the conductive properties of melanin, to which we will now turn.

**Figure 9:**
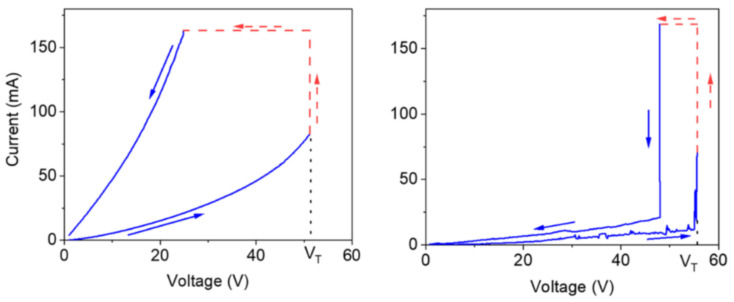
Electrical switching behavior observed in a pressed powder pellet of melanin as published by the Santanto group. On the left the behavior of an apparent dry sample is shown and on the right a wet sample is shown. As can be seen, as the voltage is increased up to a threshold voltage V_T_, the material enters a low resistance phase. Reprinted with permission from M. Raeli, A. Gouda, J. Bellemare et al., *ACS Appl. Bio Mater.*, 2020, 3, 5244–5252 [105]. Copyright 2020 American Chemical Society.

Melanin has been viewed as an example of an amorphous semiconductor since the 1970s due to a couple of observations: its broad band optical absorbance profile indicating localized states in the gap [93], the presence of the aforementioned free radical signal suggesting electrons at the Fermi level available for conduction, semiconductor like behavior of the conductivity as a function of temperature (e.g., [80,94,95,96,97,98,99]), drop in current after photocurrent measurement indicating trapped states as well as temperature dependent photocurrent behavior [100,101] and finally the ability of melanin to demonstrate bi-stable switching behavior after an application of a high enough voltage (Figure 9) [102,103,104,105] (a key characteristic of an amorphous semiconductor [7]). As such, melanin has been investigated in semiconductor related devices.

**Figure 10:**
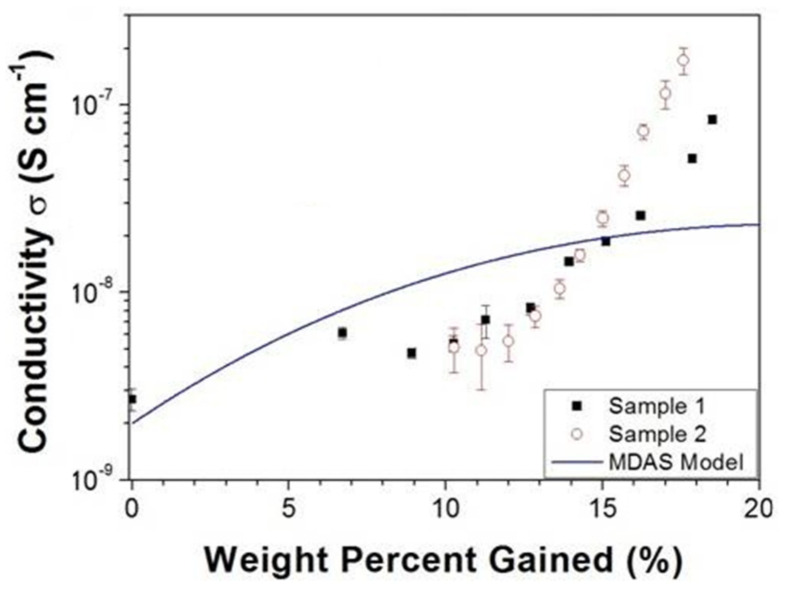
An example of the hydration dependent conductivity of melanin at room temperature. Shown are two different samples as well as a theoretical curve that represent the amorphous semiconductor model. Reproduced from A.B. Mostert, B.J. Powell, I.R. Gentle, P. Meredith, *Appl. Phys. Lett.*, 2012, 100, 093,701 [106], with the permission of AIP Publishing.

One thing that is interesting about all the observations above, is that melanin’s conductivity in the condensed matter state can be significantly changed depending on its water content (see Figure 10) [77,80,99,106,107,108]. Indeed, the switching behavior of melanin could not be induced in the dry state [107], though recent work suggests that it can (i.e., Figure 9, left) [105]. The need for hydration in obtaining switching behavior has originally led to a model where water is required to increase the dielectric constant of the material, which would narrow the band gap between the semiconductor valence and conduction mobility edges [102,106,107]. This model was tested (see Figure 10) and found to be incompatible with the experimental data [106].

The aforementioned incompatibility with theory, other hydration dependent conductivity results and muon spin resonance work has led to an alternative charge transport model for understanding melanin’s conductivity [107]. This alternative model posits that melanin’s conductivity is instead regulated by the comproportionation reaction (Figure 2). By adding water, the equilibrium shifts towards the production of both radicals and protons, which would make melanin, in principle, a mixed electronic-ionic conductor. This model has a lot of explanatory power as well. When it comes to the absorbance spectra, it relies on the chemical disorder model for explanation. The radical presence is explained by basic indolequinone chemistry. The photoconductivity behavior was also shown to be hydration dependent [106], and hence, trap states may not be the preferred explanation but that water desorption due to localized heating causes a drop in available charges. Furthermore, the temperature dependence can also be explained by the same equilibria, since equilibrium chemistry also exhibits Arrhenius, semiconducting like behavior [99]. This model has been bolstered by kinetic isotope work utilizing D_2_O hydration [89] and demonstration of photo induced semiquinone production alongside photoconductivity [92]. Proton conductivity has also been demonstrated [108], with protons apparently being the dominating charge carrier when hydrated [109], though suggestions of the presence of proton conductivity has a long history [77,80]. Finally, this model has been utilized, successfully, in creating copper doped melanin systems to enhance proton conductivity [88]. Again, it should be mentioned, that there are differences between natural and synthetic samples of melanin. However, the work presented here should indicate the broad outlines of our understanding of the pigment’s conductivity.

At this point though, it is clear from the literature that many researchers accept the presence of protons in the hydrated state, most likely due to the reaction depicted in Figure 2, but that some authors still believe that melanin’s conductivity should be explained by combining both the mixed proton-electronic model with the amorphous semiconductor model, e.g., Reali et al. [105,110]. There is clearly still scope for further research elucidating melanin’s charge transport properties and underlying mechanisms to settle which is the most appropriate charge transport model for melanin.

Melanin is also known to bind/chelate metal ions within its structure to multiple binding sites. The list of metal ions that can bind is quite impressive, including a wide variety of the alkali metals, alkali earth, transition metal ions as well as the lanthanides [23,87,111]. What is even more interesting is that the quantities and binding strengths of melanin to many ions can be quite large. For example, melanin can bind to Mg(II), Ca(II), Sr(II) and Cu(II) to 5, 4, 14 and 34 times stronger than ethylenediaminetetraacetic acid (EDTA) at pH 5.8 [112]. Thus far the binding constant for Fe(III) has not been reliably determined. For Ca(II), Mg(II) or Fe(III) the saturation levels of binding equates to around 3–4 monomer units per ion or ~1.00 mmol g^−1^ of melanin [112]. For Fe(III) this equated to around 8.0% by mass of melanin. Similar values can be obtained for Cu(II) [88] and Zn(II) [113]. Again, whether one has a natural or synthetic melanin is an important consideration. The majority of the work cited above is for naturally occurring samples. It should also be noted that the fascination of metal binding to melanin is an old one, with decades of work done on the interaction of the radical in melanin with metal ions [7]. The above should indicate that the potential of melanin to be modified or adapted with other entities is enormous for technological applications [88].

Similar to its ability to bind ions, melanin is known to bind and accumulate drugs within its structure, with this property known since at least the 1960s. For examples, see references [39,114,115,116,117,118,119,120].

It should not be surprising that melanin is a redox active material. The aforementioned metal ion studies have clearly indicated that such is the case. As a material, melanin’s redox behavior is to a large extent determined by the redox properties of its underlying monomer units, i.e., an *ortho*-quinone system [16]. For example, the redox potential of melanin as a function of pH is around 48.9–62 mV pH^−1^ [121,122,123], which is in line with the underlying thermodynamics of the monomer units where it is expected to be 59 mV pH^−1^ [17].

However, there are significant differences between the polymer material and the free monomer units. The monomer units are highly reactive and unstable due to their *ortho*-quinone and -semiquinone nature [18,20], the melanin subunits are stable and less reactive, as demonstrated by the presence of a stable free radical mentioned above. This is likely due to the melanin moieties being restricted within the polymer, barring easy access due to steric hindrance and intramolecular interactions [7]. One cannot rule out changes in ionization potentials either as a result.

There are also some other differences. For example, it appears that melanin has a one electron reduction potential of around −500 ± 50 mV versus normal hydrogen electrode (NHE) as reported by Różanowska et al. [64], whereas the closest estimated reduction potential for the monomer system is approx. −100 mV versus NHE for the reduction of deprotonated semiquinone to deprotonated catechol [17]. Różanowska et al. also estimated 25% of the sites within melanin are available for reduction, while 75% are available for oxidation. However, this is at odds with other works that suggest that the majority of the polymer after synthesis is already oxidized [124]. The above difference may simply be due to variations in the material, but given that melanin is synthesized under oxidation conditions, one would expect a mainly oxidized polymer. More systematic work is required. Other differences were also seen by Serpentini et al. where a two-electron oxidation process was observed with the corresponding potentials being +460 and +525 mV vs. saturated calomel electrode (SCE) [125]. Their data also indicated a reduction of melanin at +20 and −355 mV vs. SCE at a pH of 5.6. For comparison, the two electron reduction potentials for the monomer system for quinone to deprotonated catechol is expected to be 40 mV vs. NHE (or −210 mV vs. SCE) and for the quinone to protonated catechol is expected to be 700 mV vs. NHE (or 459 mV vs. SCE) [17]. Interestingly, Serpentini et al. did not observe any changes of the peak potential with pH in the pH range 2–8, which is odd considering that melanin’s functional groups exhibit distinct changes of their protonation state in these pH ranges [7]. Finally, oxidative polymerization of DHI (a close relative to melanin) was also studied using direct electrochemistry [126], where it was concluded that the oxidation half-potential was +125 mV vs. the Ag–AgCl electrode and attributed to the formation of the quinone–imine.

### 3.3. A Functional, Flexible and Versatile Biomacromolecule

When it comes to flexible polymers for technological application, the term “flexible” can be used in two senses: versatility or mechanical suppleness. In this section both aspects will be explored by inspecting some of the technological settings in which melanin has been applied. This section will show how the above physico-chemical properties of melanin have been employed, which may serve as inspiration to the potential researcher to develop their own applications for melanin as a flexible polymer material.

Utilizing melanin’s unique absorbance profile, which increases with increasing photon energy, melanin has been used as a coating for lenses to filter out high energy photons [127]. Indeed, there are commercial enterprises to this effect, for example Photoprotective Technologies, Inc. [128] (whose CEO, Dr. Gallas, was a well-known melanin researcher) is a company that produces melanin coated glasses for various applications including sunglasses and blue-blockers (glasses that aid sleeping by blocking blue light).

The unique absorbance profile of melanin has also been tested in medical settings directly related to application of the human skin. For example, melanin mixed with keratin has been used to make a “second skin”, where the melanin acts as a pigment to adjust to human skin tone [129].

Another example of utilizing melanin’s optical properties is its use as a colorant, which is achieved by also manipulating its structural properties via the use of melanin nanoparticles deposited on a substrate [130,131,132]. Fascinatingly, colors can also be changed via the use of hydration, which is due to changes in the refractive index of the melanin as the nanoparticle films swell [133].

For recent, in-depth reviews about this structural colorant approach to using melanin, Xiao et al. and Kohri et al. are highly recommended [76,134]. These authors also discuss the optical properties of melanins as well as applications including UV protection agents, fluorescent probes and photothermal therapy and as such will not be reviewed here.

Melanin has also been reported as a potential E-ink, showing excellent resolution [135].

**Figure 11:**
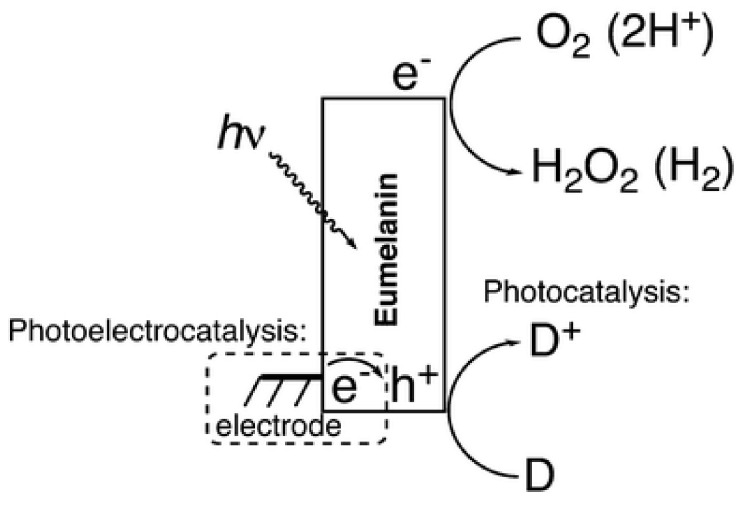
The proposed mechanism for DHI-melanin’s photocatalytic effect. Light is absorbed by melanin where electronic charges are generated. Reduction of oxygen to hydrogen peroxide proceeds in oxygenated conditions, or reduction of protons to hydrogen in deoxygenated conditions. Figure adapted with permission from L. Migliaccio, M. Gryszel, V. Ðerek et al., *Mater. Hor.*, 2018, 5, 984 under Creative Commons Attribution—Published by The Royal Society of Chemistry [136].

In keeping with the theme of utilizing the optical properties of melanin, a recent work by Migliaccio et al. demonstrated that a DHI based melanin could be made to act as a photo catalyst (Figure 11) [136]. They were able to show that across the whole absorbance spectrum, melanin could be made to produce peroxide, hydrogen and cause photo oxidation of various organic substances. In essence, melanin could be utilized in the production of hydrogen, a potential future energy source.

**Figure 12:**
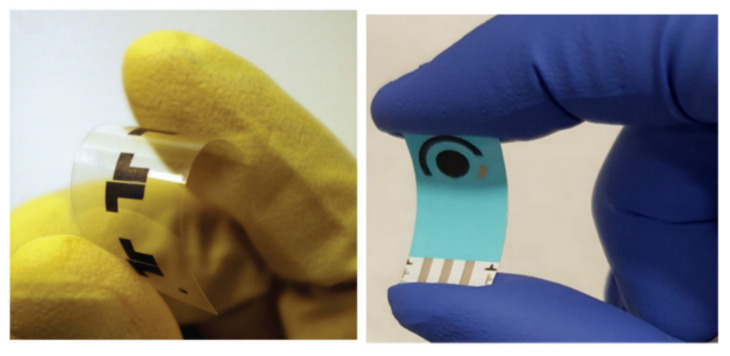
(**Left**) A real life image of micro-supercapacitors on a flexible polyethylene terephthalate (PET) substrate. Reproduced from [137] with permission from The Royal Society of Chemistry. (**Right**) A real life image of a pH sensor using melanin on a polyethylene (PE) substrate. Reproduced with permission from Tehrani et al. [121] under Creative Commons Attribution—Published by IOP Publishing.

Utilizing the proton generation capability of melanin and its redox properties, and demonstrating its mechanical flexibility for device applications, Kumar et al. showed that melanin can be made into a flexible supercapacitor (see Figure 12, left) [137]. They reported a high specific capacitance of 24 mA h g^−1^ with a maximum power density of up to 20 mW cm^−2^. The high capacitance is supported by impedance work that showed that hydrated melanins can have a real dielectric constant of ~10^3^ at low frequencies, which is far beyond the dielectric constant of water and is likely due to the high proton concentration [109]. This exciting development by Kumar et al. demonstrates that melanin has the potential to be made into flexible supercapacitor electrodes. Furthermore, it suggests that melanin can act as a high dielectric material.

Continuing on the topic of melanin’s redox capacity and ability to bind ions, the body of work by the Bettinger group is an excellent example of the capabilities of melanin. For example, melanin was made into an energy storage device by loading it with sodium ions with specific capacities of 30.4 ± 1.6 mA h g^−1^ [138]. They have also created cathode electrodes utilizing melanin’s capabilities to bind Mg^2+^ [139]. All their work has led to a patent for a water activated ingestible battery for powering medical devices [140]. Finally, they have also shown that melanin can be used as an ionic “filter”, to separate out ions from one another, for example Mg^2+^ and Li^+^ from a brine [141].

Another example of utilizing melanin’s redox properties and its mechanical flexibility was produced by Tehrani et al. (see Figure 12, right) [121]. In this work they demonstrated a very sensitive and stable electrochemical pH sensor by depositing a sulfonated melanin (see below) onto graphene and a flexible polyethylene (PE) substrate. This pH sensor showed a potential/pH dependence of 62 ± 7 mW pH^−1^ in the range of physiological pH (5–8), which is one of the highest recorded [121]. Similar work has been pioneered by the Graeff group where they created an electrochemical pH sensor utilizing an extended gate field effect transistor, where they observed a potential/pH dependence of up to 48.9 mW pH^−1^ across a pH range of 2–12.

**Figure 13:**
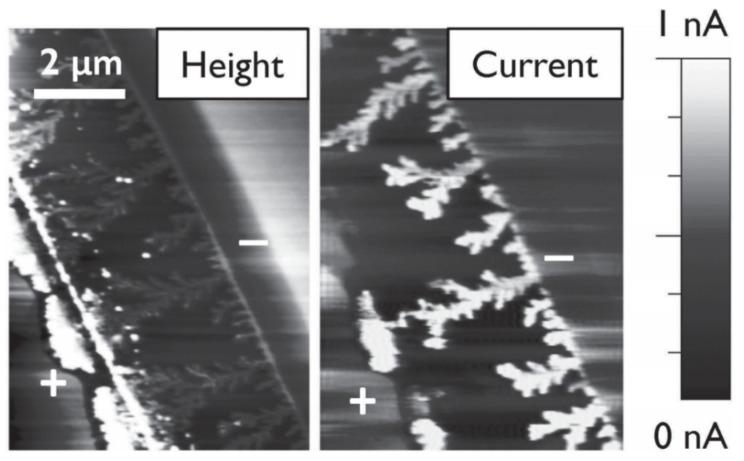
An example of an atomic force microscopy (AFM) image of melanin films and the beautiful gold dendrites. On the left is an image measuring the height of the film and on the left is a conductive AFM image. Reprinted with permission from Wünsche et al. [142]. © 2013 John Wiley & Sons, Inc. (Hoboken, NJ, USA).

An interesting and accidental discovery was made by Wünsche et al. where they tested melanin thin films on gold electrodes under humid conditions [142]. They found that, under long periods of voltage bias, one is able to form gold dendrites (Figure 13), which form at the positive electrode and grow towards the negative electrode. They could achieve this effect as long as melanin had phenolic hydroxyl groups present. It is believed that this effect of the dissolution of the Au electrode is enabled by low amounts of Cl^−^ present in melanin coupled with its reducing ability and metal ion binding capability. This result could potentially be exploited for the in situ formation of Au-melanin nanostructures and biocompatible resistive switching memory devices.

**Figure 14:**
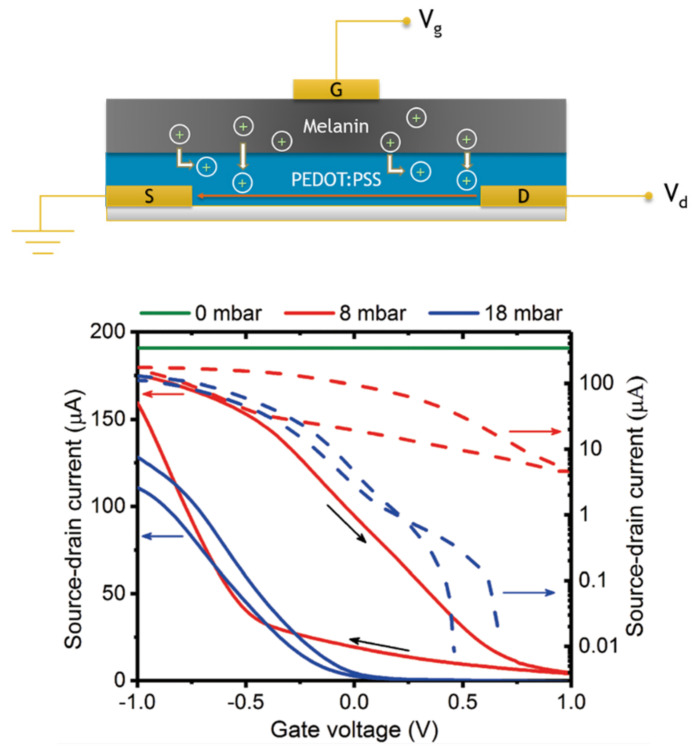
(**Top**) A schematic of the solid state organic electrochemical transistor from Sheliakina et al. The three terminal device is made of a bilayer utilizing poly(3,4-ethylenedioxythiophene) polystyrene sulfonate as the conductive channel and melanin as the ionic gate. (**Bottom**) Example transfer curves for the devices demonstrating on/off regimes as a function of an in situ hydration control. As can be seen, proper transistor function and sensitivity is recovered with hydration. Adapted with permission from M. Sheliakina, A.B. Mostert, P. Meredith, *Mater. Hor.*, 2018, 5, 256 under the Creative Commons Attribution—Published by the Royal Society of Chemistry [143].

Explicitly utilizing the comproportionation reaction and hydration dependence, Sheliakina et al. were able to demonstrate the first ever all solid state organic electrochemical transistor based upon a biomaterial (i.e., melanin), see Figure 14 [143]. The interesting thing about these transistor devices was that, as they were wetted, their performance improved. This is mainly due to increasing water content leading to an increase in protons via the comproportionation reaction (Figure 2). The device works by injecting protons from the melanin top layer into the conductive polymer channel, where the protons de-dope the channel. Given that melanin is mechanically flexible and biocompatible, this work demonstrates that bioelectronic sensing devices based on melanin for potential in vivo applications can be a reality, as opposed to many other conductive materials whose performance degrades with hydration.

As mentioned in the introduction, there is a need for biomaterials to create low-cost devices with a reduced environmental footprint. Hybrid devices incorporating biomaterials into traditional semiconductor systems will form part of the solution. The first such example utilizing melanin was published by Ambrico et al. where the authors created a metal-insulator-silicon device using melanin as the insulator material [144].

**Figure 15:**
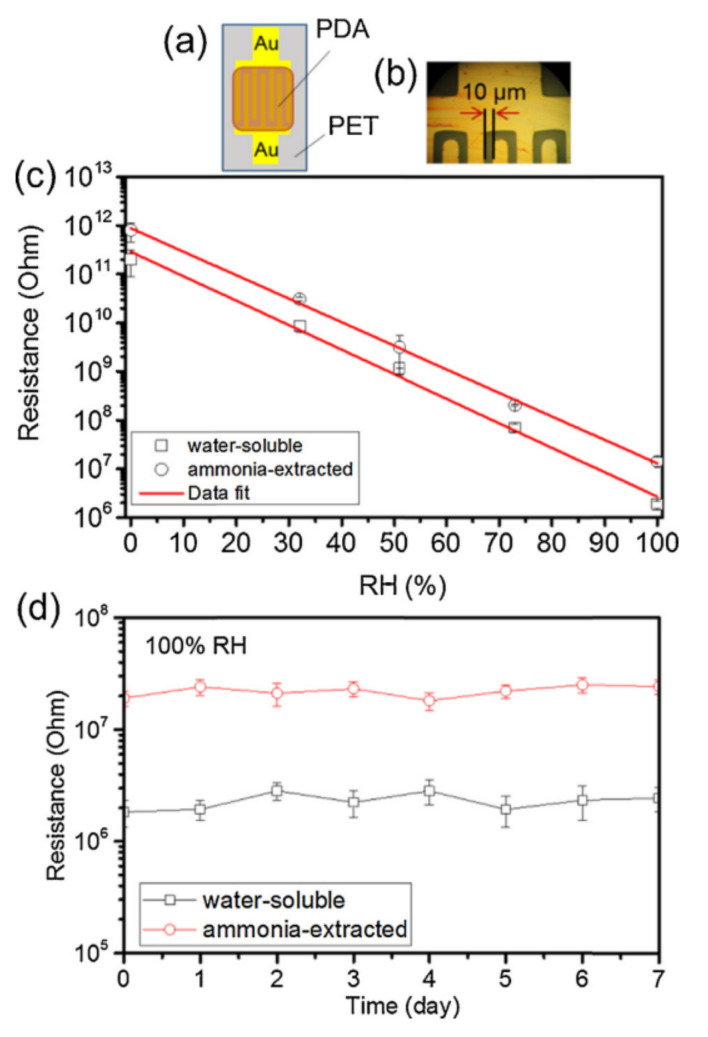
(**a**) A schematic structure of the humidity sensing device that Wu et al. fabricated from a polydopamine (PDA) melanin. (**b**) Shows an optical image of the device. (**c**) The resistance vs. relative humidity is plotted for two different variations of melanin films, one based upon a water-soluble polymer and the other based upon an ammonia extracted procedure. As can be seen, there is an exponential dependence on the resistance with humidity, making the device sensitive to moisture content. (**d**) An example of the resistance measured over days, indicating a stable device. This figure was published in Sensors and Actuators B: Chemical, 224, T.F. Wu, J.D. Hong, Synthesis of water-soluble dopamine–melanin for ultrasensitive and ultrafast humidity sensor, 178–184, Copyright Elsevier 2016 [145].

Naturally, melanin’s hygroscopic nature and its hydration dependent conductivity would suggest that it would make a natural choice as a sensing element in a humidity sensor. Wu et al. demonstrated that a fast, stable and accurate humidity sensor can be made from a polydopamine melanin (see Figure 15) [145,146]. This sensor was fabricated on a polyethylene terephthalate (PET) substrate, but the authors did not demonstrate any mechanical testing.

Melanin has also been incorporated into organic voltaic devices, instead of covering it here, the reader is directed to a recent review by Vahidzadeh et al. [147].

In line with melanin’s drug binding capabilities, melanin nanoparticles have been tested for biomedical applications. A recent, large review going into such detail has been published by Caldas et al. [148]. As such, an in-depth discussion will not be covered here and the interested reader is directed there.

Another large field of study is the testing of melanin as a part of regenerative medicine. Melanin and melanin-mimicking material coatings have demonstrated enhanced cell attachment and the ability to stick to various substrates. Furthermore, it can enhance healing in skin, bone and nerve defects in model in vivo systems. For an excellent, recent review on this aspect of melanin materials, please consult Cavallini et al. [149].

Finally, the reader may be interested in exploring work that focuses solely on naturally sourced melanins. As such, a recommended review is by Xie et al. [150], where the authors discuss applications developed from naturally occurring melanin and contrasting work with polydopamine.

## 4. How to Start Work on Melanin

### 4.1. Novel Synthetic Methods for Materials Scientists

Having hopefully demonstrated that melanin is an interesting, flexible and versatile polymeric material, the interested researcher may be drawn to investigate the material for applications, or more fundamental purposes. However, a natural question to ask is where to begin?

There are essentially two classes of melanin, or eumelanin, to consider. There are natural sources and then there are synthetic sources. Naturally, the aim of the researcher will determine which class is suitable, and both classes have their advantages and disadvantages. An excellent article to consult on these matters, which also includes examples of how to obtain melanin, both eumelanin and pheomelanin, is written by d’Ischia et al. as mentioned above [11]. In their article they list the various sources of natural melanin and the basics of extraction. They also highlight various sources of synthetic melanin and their procedures for synthesis, which should prove useful to researchers wanting to investigate various version of the polymer. d’Ischia et al. wrote other articles highlighting properties of melanin (and also contrasting to polydopamine), but made a big emphasis on the different effects of DHI and DHICA ratios, and as such they are useful summaries to that effect and should be considered by researchers in their materials synthesis [39,55].

Given that this review is focused on material applications, the most likely route for the polymer or materials scientist would be to make a synthetic biomimetic of melanin. To get the interested scientist started, one of the simplest methods for materials synthesis was first described by Felix et al. [23]. This method allows for the synthesis of a cheap product, that is a biomimetic of melanin and should enable a materials scientist to “get going” on doing work on melanin. In brief, 5–10 g of dl-dopa (dl—dihydroxyphenylalanine) (obtained from Sigma for example) is dissolved in water, adjusted to pH 8 and then the solution is stirred for 3 days while air is bubbled through the solution. The material is then extracted via a precipitation by adjustment of the solution to pH 2 (e.g., using HCl) and filtered and washed. This will yield a system that is dominated by DHI content, with around 10% of moieties being DHICA [11,56,57], but is an excellent biomimetic.

Electrochemical methods can also be employed, which may be of interest to researchers who want to create free standing films. For example, Subianto et al. developed a method to synthesize a free-standing melanin onto an indium tin oxide (ITO) substrate by passing a current through a solution of dl-dopa in water held at a pH of 9 using a buffer [151]. Another such example is work by Kim et al., where the authors synthesized a melanin analogue, poly(5,6-dimethoxyindole-2-carboxylic acid) (DMICA), by electrochemically depositing the material on a stainless steel mesh by passing current through a DMICA solution in acetone [138]. The films were then mechanically delaminated.

Another method of synthesizing melanin, while depositing it on a substrate, is to take the approach of polydopamine deposition [152,153], (noting that polydopamine is a close analogue to melanin), as Kwon et al. [154] and Park et al. did [141], simply by leaving the substrate in the solution as the material is being synthesized per normal methods. Li et al. have extended this method to L-dopa [72], making a “truer” melanin with this methodology.

A third methodology for creating melanin or melanin like films on a substrate in situ is utilizing the ammonia-induced solid-state polymerization (AISSP) technique developed by Pezzella et al. [155]. In this method, a DHI melanin was made by spin coating the DHI monomer (in methanol) onto a substrate that was subsequently annealed. The film was then exposed to air containing ammonia vapor, which would then allow polymerization to occur.

Other new melanin synthesis methods have also been developed, specifically with the aim to enable melanin to be used in materials applications, which should prove of great interest to material and polymer scientists. For example, instead of synthesizing melanin in water as per most common techniques, one can synthesize in another solvent to enable better solubility of the final product. This has been the approach of the Graeff group. They demonstrated that melanin can be made in dimethyl sulfoxide (DMSO) and *N,N*-dimethyl formamide (DMF), yielding spin coated films with a mean square roughness of ~0.3 nm [156]. They have further investigated the melanin in DMSO procedure (which they call D-melanin) [157], and determined that additional moieties within melanin are incorporated. Essentially, some indolequinone units have additional sulfonated groups at the 2, 5 and 6 positions, in various proportions. The great advantage of this melanin is that it can be spin coated out onto any substrate, including hydrophobic substrates, and is stable. Furthermore, it can be spin coated out of other solvents if need be, such as DMF and 1-Methyl-2-pyrrolidone (NMP) [158]. One drawback to this approach is the long synthesis times, which can take up to 3 weeks. Furthermore, a question can be asked as to whether the sulfonation precludes the material as a melanin. Recent magnetic resonance work suggests that the free radical nature of the D-melanin is quite similar to the standard melanin, barring the presence of a sulfonated radical caused by degradation and more hydrophobicity [91]. Electrochemical work also shows an expected potential/pH gradient consistent to that seen for melanin [121]. Thus, indications are that these materials are a melanin (though not a true biomimetic), with the additional bonus of spin coating these polymeric materials on a wide array of substrates. However, it is yet to be seen how their electrical, optical and structural properties compare to a “standard” melanin, though indications are present that it also sustains a proton current [159].

The Graeff group have also pioneered other methods of synthesis, again to enable the ease of processing for materials applications. They have synthesized the above D-melanin under O_2_ pressure, creating a polymer with a greater carboxylated content and improving the reaction rate [160]. This idea of synthesizing melanin under O_2_ pressure has also been applied to melanin synthesis in water [161]. The resultant melanin does have structural differences to a standard melanin, most notably higher carbonyl content and different surface charges. It has the great advantage of making the material more soluble in water. However, like its DMSO cousin, it is yet to be seen how their electrical, optical and structural properties compare to a “standard” melanin.

### 4.2. Novel Post Fabrication Techniques for Material Applications in Devices

The above-mentioned reviews do not contain much information when it comes to post synthesis fabrication techniques for materials applications. Given that this article is written more towards polymeric and other materials scientists, an overview to some of these techniques will be given here.

The simplest approach to making a melanin sample for study is to take a powder of the material, and press them into pellets using a hydraulic press (see for recent example references [88,92,99,105,158]). This method is still popular for studying the more fundamental properties of melanin than for actual device applications. These pellets can then be contacted or tested spectroscopically. Naturally, these pellets are fragile and not conducive to devices.

When it comes to making melanin in to a material for device applications, a central issue in dealing with melanin is the above-mentioned insolubility in many common solvents, as may be inferred in our discussion above on the novel techniques of melanin synthesis. As such, making a melanin “solution” to enable coating require various methods. One such method was published by Bothma et al. [48]. Reported therein, the authors re-suspend a synthetic melanin powder in a highly alkaline solution using ammonia, which is sonicated and then used to spin coat onto hydrophilic substrates. The result is homogenous, device quality thin films of melanin. The great advantage of this method is that the ammonia evaporated off, allowing the melanin to partially precipitate while the pH is still high enough to allow the solution to spread across a substrate. The big downside to the method is that the high pH environment may in fact be modifying the chemical structure of melanin somewhat, by causing aromatic ring fission [162]. Evidence for this has come from x-ray photoelectron spectroscopy (XPS) measurements showing a relative increase in nitrogen concentration [79,158]. A similar approach has been used by Ambrico et al. [144]. For these above methods to work, the substrate has to be hydrophilic, and hence these authors, and many others in the literature, go to great lengths to prepare their substrates accordingly. In essence, if one can take a substrate, such as silicon/silicon dioxide, and create hydroxyls on the surface, the substrate will be wetted by these solutions and enable spin coating.

As an alternative to using water, another way to create a melanin suspension/solution for creating films was pioneered by Abbas et al. [98]. By dissolving an already made synthetic melanin powder in a mixture of DMSO and methanol, they were able to create a suspension that can be sprayed onto a substrate (held at high temperature), creating films that are uniform with controllable thickness.

The approach of using different solvents to suspend melanin in to enable spin coating is quite widespread, with DMSO and DMF proving popular, for example see Wünsche et al. [108,142], with a concomitant cleaning and preparation of substrates. This method was also used by Kumar et al., who used DMSO to suspend the melanin and then utilized a drop cast technique to deposit the melanin onto a substrate, followed by driving off the solvent under vacuum [137].

Melanin can also be prepared in the form of electrodes, by combining it with another material as a binder or reference. For example, Serpentini et al. combined melanin with carbon paste [125], whereas Kim et al. used polytetrafluoroethylene (PTFE) as a binder [139].

Finally, one more notable method for creating melanin samples for testing was by Xiao et al., who have made colored melanins (mentioned above) [130,133]. Their method for making melanin is more along standard lines, though they do use ethanol with water and ammonia. As an aside, the exact ratios of solvents to material determines the size of melanin particles produced, which is critical to their endeavors. What is of interest here is their deposition method. Essentially, a melanin film can be deposited by dipping a prepared substrate into a hot melanin solution/suspension (in their case 60 °C), with the solution allowed to evaporate overtime, letting the melanin deposit on the surface as the solution level drops.

### 4.3. A Note on Characterization

As has been made clear above, melanin is insoluble in most solvents [11]. This makes basic chemical characterization a difficult task. As such, the approach of most of the works cited above is to characterize their samples using macroscopic observables such as UV-Vis, X-ray Photoelectric Spectroscopy (XPS) and EPR due to their relative ease.

UV-Vis absorbance testing of product partially dissolved in moderate alkaline conditions is an excellent method for characterization since the absorbance spectrum is due to the chromophore and not scattering. Though one has to take note that melanin derivatives may not quite show the smooth exponential dependence of the spectrum depicted in Figure 7. It should be noted that for thin films of melanin, also deposited out of other solvents, show the same UV-Vis behavior as for dilute solutions and hence can be applied to post fabricated samples as well [11].

EPR, especially a basic X-band spectrum will also be a good characterization of a melanin sample. The example spectrum shown in Figure 8 is a well-known observation almost universal across both natural and synthetic samples and some derivatives [7], and as such is a good characterization method.

Since elemental analysis is made difficult due to insolubility, XPS has proven popular as an elemental test. It can be applied to films of material as well pressed powder samples, it is convenient and done under vacuum, which drives off physisorbed water that may otherwise skew the elemental composition. However, only recently has depth profiling XPS been performed on various melanin polymers to demonstrate that XPS surface scans are a representation of the bulk elemental composition [158]. Paulin et al. showed that on basic elemental composition, for pressed powders and thin films, the surface XPS scans yielded the same information as for the bulk [158]. When it came to the detailed chemistry, pressed powders showed very similar chemistry between the surface and the bulk, whereas for films, the chemistry appeared to be exactly the same. As such, a standard surface XPS scan appears to be the go-to method for elemental analysis.

## 5. Conclusions

Eumelanin, commonly referred to as melanin, is a polymeric material that has captured the interests of various scientists for at least a century. This is due to its unique physical and chemical properties, which are still not well understood, nor is its biological function fully comprehended. Due to its complexity and properties, melanin has shown much potential as a functional polymeric material. In this review, melanin’s structure, properties and a wide variety of example applications have been covered to demonstrate to the materials scientist that melanin is a prime candidate for investigation for functional applications. Furthermore, also reviewed were initial ways a new researcher to the field can begin synthesizing and fabricating melanin samples for testing, especially for device and sensing applications. The material should also be of interest to other scientists as well as engineers, and as such, melanin should prove to be a next generation bio polymer for solving medical and electronic waste problems of modern society.

## Data Availability

The data presented in this study are available on request from the corresponding author.
